# Self‐enhancement and physical health: A meta‐analysis

**DOI:** 10.1111/bjso.12577

**Published:** 2022-09-06

**Authors:** Ethan Zell, Christopher A. Stockus, Tara L. Lesick, Constantine Sedikides

**Affiliations:** ^1^ UNC Greensboro Greensboro, North Carolina USA; ^2^ University of Southampton Southampton UK

**Keywords:** comparative judgement, health, narcissism, physical health, self‐enhancement

## Abstract

A prior meta‐analysis yielded a positive relation between self‐enhancement and psychological health. We present the first meta‐analysis of the association between self‐enhancement and physical health (*k* = 87; *N* = 22,415). We relied predominantly on social desirability as an operationalization of self‐enhancement and secondarily on comparative judgement and narcissism. Further, we operationalized physical health in terms of self‐rated health, symptoms and biomarkers. Overall, self‐enhancement yielded a near‐zero association with physical health, *r* = .01. However, this association was more pronounced for comparative judgement (*r* = .18, *k* = 6) than social desirability (*r* = .03, *k* = 41) or narcissism (*r* = −.0001, *k* = 8), and for self‐rated health (*r* = .09, *k* = 9) than symptoms (*r* = .01, *k* = 29) or biomarkers (*r* = −.13, *k* = 17). The association between self‐enhancement and physical health fluctuates across measures of both constructs calling for more focussed and nuanced investigations.

## BACKGROUND

People frequently evaluate themselves in formal and informal settings. For example, people evaluate their performance at school and work, their intelligence after taking a standardized test, their attractiveness after glancing at themselves in a mirror, their social skills after a gathering with family and friends, their personality after taking an online survey and their health and wellness after a medical screening. Hundreds of studies have examined self‐evaluations across a variety of attribute and ability dimensions. These studies yield a consistent conclusion: self‐evaluations on most dimensions—especially ones that are personally important—are more favourable than external indicators suggest they should be (Dunning, 2015; Gebauer et al., 2013; Zell et al., 2020). In contemporary research, the tendency to have unduly positive self‐views is referred to as *self‐enhancement* (Baumeister, 1998; Marshall & Brown, 2008; Sedikides & Strube, 1997). Although most people self‐enhance at opportune times, self‐enhancement reflects a stable individual difference that ranges from self‐criticism to self‐aggrandizement (Hepper et al., 2010, 2013; Kwan et al., 2004; Paulhus, 1998).

As of present, there is little debate about the existence of self‐enhancement both in Western and Eastern cultures (Brown, 2010; Chiu et al., 2011; Sedikides et al., 2015). Several reviews catalogue the many ways in which people manifest unduly favourable self‐views (Alicke et al., 2020; Alicke & Sedikides, 2009; Brown, 2007; Sedikides, 2020; Shepperd et al., 2015). Further, self‐enhancement has been positively associated with psychological health (Dufner et al., 2019; Segerstrom & Roach, 2008; Taylor & Sherman, 2008), with this association being partly heritable (Luo et al., 2020), and confers psychological health (Dufner et al., 2015; O'Mara et al., 2012; Zuckerman & O'Loughlin, 2006). It is not clear, though, whether self‐enhancement is also positively associated with, or confers, physical health, as reflected in self‐rated health, symptoms or diseases and biomarkers. We present the first meta‐analysis of research on the association between self‐enhancement and physical health.

### Self‐enhancement

Several operationalizations of self‐enhancement exist in the psychological literature (Alicke & Sedikides, 2011; Sedikides & Gregg, 2008). For example, in one empirical stream, participants judge themselves in comparison to an average peer; here, they often evaluate their abilities, attributes and future prospects as above average despite this being improbable or logically impossible in many circumstances (Alicke & Govorun, 2005; Logg et al., 2018; Sedikides et al., 2014). In another empirical stream, participants' self‐evaluations are compared with external criteria, such as peer assessments, expert assessments or scores on objective tests; here, participants often evaluate their attributes, abilities and personality more favourably than external indicators suggest they should (Dufner et al., 2012; Gregg et al., 2011; Preuss & Alicke, 2009). In yet another stream, researchers operationalize self‐enhancement via relevant individual difference variables. One is grandiose (and in particular, agentic) narcissism, which is characterized, in part, by inflated self‐views and pomposity (Grijalva & Zhang, 2016; Sedikides, 2021a; Sedikides & Campbell, 2017); indeed, narcissism has even been labelled ‘the self‐enhancer personality’ (Morf et al., 2011, p. 399). The other individual difference variable is socially desirable responding, which also reflects inflated self‐views (Hart et al., 2015; Paulhus, 2002); indeed, ‘In the context of questionnaire styles, self‐enhancement is typically referred to as socially desirable responding’ (Paulhus & Holden, 2010, p. 227).

Self‐enhancement is different from related constructs such as self‐esteem and optimism, which refer to the tendency to have positive views of the self or the future (Shepperd et al., 2015; Zell et al., 2020). Measures of self‐esteem and optimism capture the valence of self‐views and future beliefs, that is, whether they are positive, negative or neutral (Davidson & Prkachin, 1997; Leary & Baumeister, 2000). Conversely, self‐enhancement reflects the tendency to have self‐views or expectations for one's future that are *positively biased* and thus deviate from reality in a self‐flattering direction. Thus, a person with high self‐esteem who generally evaluates themselves favourably may not necessarily be doing so in error. Similarly, optimistic expectations about one's future may sometimes be accurate estimates of a prosperous future. Clearly then, although self‐enhancement is often correlated with self‐esteem or optimism, these constructs are independent of each other. Indeed, evidence suggests that self‐enhancement may have divergent associations with psychological adjustment and health than self‐esteem or optimism (Konrath & Bonadonna, 2014; Sweeny & Andrews, 2017). Given the differences between these constructs, and that prior reviews have given more attention to the associations between self‐esteem (Antonucci & Jackson, 1983; Baumeister et al., 2003; Miller & Downey, 1999) and optimism (Rasmussen et al., 2009; Scheier & Carver, 2018) with physical health, we focus exclusively on self‐enhancement.

### Self‐enhancement and psychological health

In addition to documenting self‐enhancement, research has been concerned with its potential benefits for psychological health. The issue has captivated scholars in social, personality, clinical and health psychology, as well as laypersons for over three decades. Taylor and Brown's (1988) landmark article on the topic, which inspired much of this work, has been cited over 12,000 times and over 6000 times since 2010 (Google Scholar, 15 August 2022).

Recently, a meta‐analysis (Dufner et al., 2019) examined the association of self‐enhancement with psychological health. Self‐enhancement was largely operationalized in terms of comparative judgement (self‐evaluations, peer evaluations), narcissism and socially desirable responding. Psychological health was operationalized in terms of life satisfaction, positive affect, negative affect and depression. The results yielded a positive association of self‐enhancement with psychological health, especially when self‐enhancement was measured via self‐evaluations (*r* = .18, *k* = 237) as opposed to peer evaluations (*r* = .12, *k* = 29).

### Self‐enhancement and physical health

Emerging research has also begun to examine the implications of self‐enhancement for physical health. However, qualitative reviews have come to different conclusions, with some suggesting that self‐enhancement is generally beneficial for physical health (Segerstrom & Roach, 2008; Taylor & Sherman, 2008), and others suggesting that it is detrimental to it (Konrath & Bonadonna, 2014; Sweeny & Andrews, 2017).

We took an exploratory approach. If self‐enhancement conduces to psychological health, then it might also conduce to physical health, given the robust connection between psychological and physical health (Schneiderman et al., 2005; Steptoe, 2019). Self‐enhancement may also conduce to physical health through another avenue. In particular, self‐enhancement is associated with goal‐pursuit and goal‐perseverance (Alicke & Sedikides, 2009; O'Mara & Gaertner, 2017; Sedikides et al., 2016), and so, high self‐enhancers may persist longer in maintaining healthy habits and avoiding health risks (e.g. obesity). Both of these perspectives anticipate a positive association between self‐enhancement and physical health. An alternative perspective anticipates a negative such association. Self‐enhancers may consider themselves rather invulnerable to physical health hazards (Jefferson et al., 2017; Shepperd et al., 2015; Zell & Sedikides, 2022), thus neglecting health check‐ups and increasing their health risks (e.g. cancer). Finally, if both positive and negative pathways from self‐enhancement to health exist, they could cancel each other out, leading to no overall association between these constructs.

The link between self‐enhancement and physical health is of considerable practical importance given its relevance to daily functioning and longevity. Moreover, unlike mental health outcomes, which are often provided through self‐report and subject to responses biases that may inflate correlations (Strickhouser et al., 2017), physical health is frequently assessed via an objective process (e.g. direct measures of biomarkers or disease diagnosis), which offers a robust estimate of self‐enhancement effects. As in a prior meta‐analysis (Dufner et al., 2019), we operationalized self‐enhancement broadly to include a variety of indices of unrealistically positive self‐views, such as comparative judgement, narcissism and socially desirable responding, as well as optimistic bias. Also, as in prior meta‐analyses (Quon & McGrath, 2014; Zell et al., 2018), we operationalized health broadly to include direct indicators of physical health including self‐rated health, physical symptoms and biomarkers.

Our meta‐analysis primarily focussed on the overall or cumulative association of self‐enhancement with physical health across populations or participant groups. In addition, we explored whether this association is moderated by several factors, including the type of self‐enhancement measure and health outcome tested in prior research, sample characteristics such as age, gender and race, as well as methodological characteristics of prior studies. Self‐enhancement measures are generally conceptualized as representing a single higher‐order construct (Dufner et al., 2019; Sedikides & Gregg, 2008; Taylor & Brown, 1988), and thus we did not expect noticeable differences in health associations across these measures. However, we anticipated that physical health outcomes measured via self‐report (e.g. self‐reported health) would be more susceptible to response biases and hence yield stronger associations with self‐enhancement than physical health outcomes measured more objectively (e.g. through biomarkers). Finally, prior work indicated that the relation between self‐enhancement and psychological health is largely constant across demographic groups (Dufner et al., 2019). Therefore, we did not expect effect sizes to vary substantially as a function of sample characteristics.

## METHOD

### Article search and inclusion criteria

We searched three relevant databases (i.e. PsychINFO, CINAHL, PubMed) for records that explicitly mentioned self‐enhancement and health in the title, abstract or keywords. Self‐enhancement search terms included narcissism, overconfidence, optimism, optimistic bias, positive illusions, self‐enhancement, self‐serving and social desirability (Dufner et al., 2019). Health search terms consisted of disease, death, health and physical health (Cundiff & Matthews, 2017). We identified additional studies by scanning the reference lists of major reviews (Alicke & Sedikides, 2009; Dufner et al., 2019; Konrath & Bonadonna, 2014; Sweeny & Andrews, 2017; Taylor & Sherman, 2008). Further, we requested unpublished studies on self‐enhancement and health from the SPSP Connect! open forum. We restricted the search to studies published or reported in English, given that English was the only language spoken by the study screeners and coders.

To be incorporated in the meta‐analysis, studies obtained in our search had to meet the following criteria: (a) include a measure of self‐enhancement, (b) include a measure of physical health and (c) provide a relevant effect size indexing the association between self‐enhancement and physical health. As mentioned above and described below, we defined self‐enhancement broadly to encompass a variety of measures indexing the degree to which individuals have positively biased self‐views (Dufner et al., 2019; Sedikides & Alicke, 2012, 2019), and we defined health broadly to encompass a variety of physical health indicators (Quon & McGrath, 2014; Strickhouser et al., 2017; Zell et al., 2018). Given our focus on self‐evaluation bias, we excluded studies that only examined the positivity of self‐views or expectations (e.g. research on self‐esteem, optimism or optimistic explanatory styles).

**FIGURE 1:**
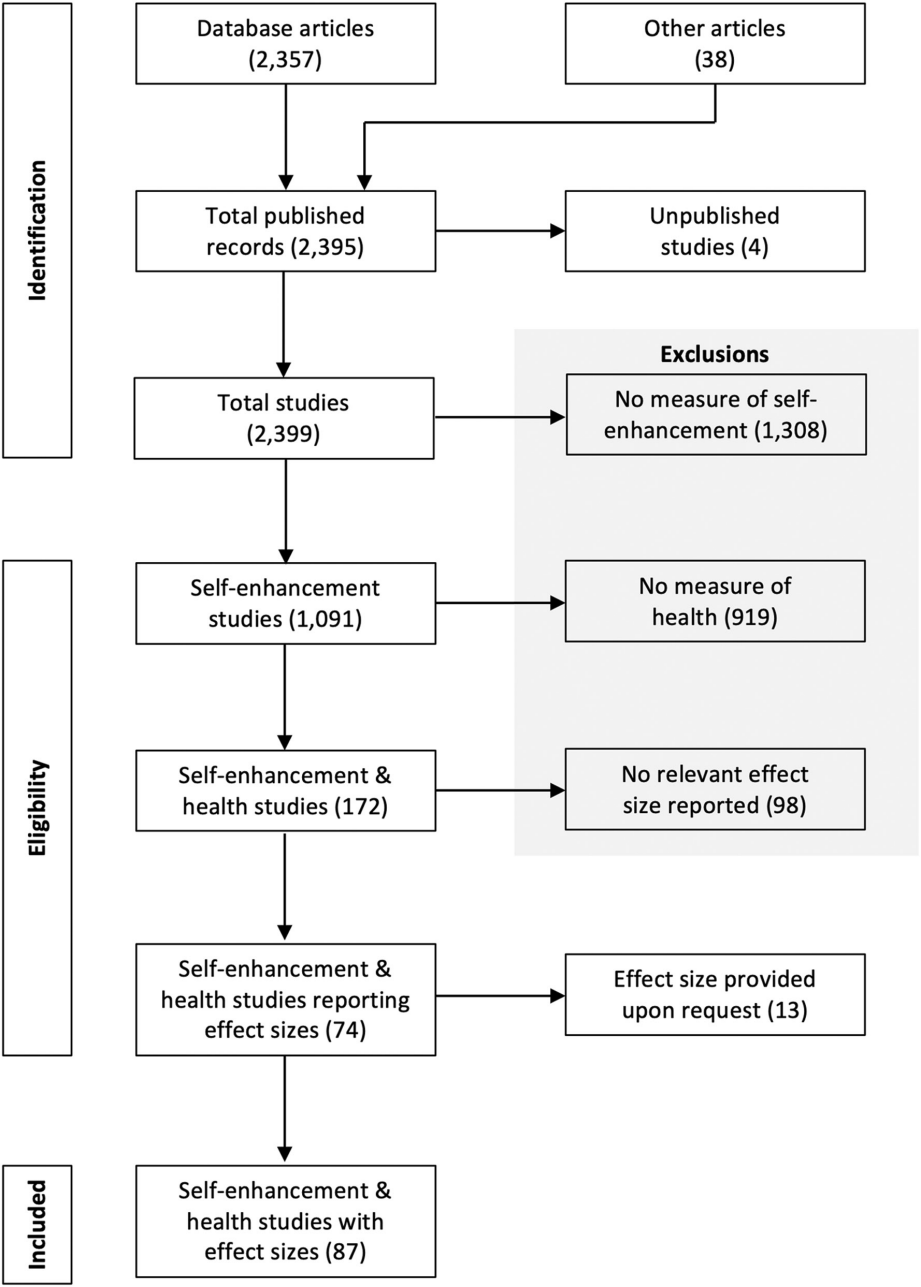
Flow chart for the article search

We screened a total of 2395 published articles and 4 unpublished studies (Figure 1). Several studies included a measure of self‐enhancement and health but did not provide a relevant effect size (*n* = 98). We contacted the corresponding author of studies published since 2005 to request the unreported effect (*n* = 65), leading to the obtainment of 13 effects (20% response rate). After exclusions, we were left with 87 studies (83 published, 4 unpublished) that collectively provided data from 22,415 participants. Each article furnished relevant data from a single study or method, and thus, each contributed a single effect to the overall model (*k* = 87).

### Effect extraction and coding

We extracted from included studies effect sizes (*r*) indexing the overall size of the relation between self‐enhancement and physical health. Most studies provided the zero‐order correlation between self‐enhancement and physical health (*n* = 56) or provided this association in an effect size metric ($\eta_{p}^{2}$ or *d*) that could be converted to *r* (*n* = 2). The remaining studies compared self‐enhancement across groups that were relatively high versus low in physical health (*n* = 18) or health across groups that were relatively high versus low in self‐enhancement (*n* = 11). In these cases, we used the provided descriptive statistics (*M* and *SD*) or statistical test information (*t* or *F*) to calculate Cohen's *d*, which we then converted to *r*.

We coded effect sizes such that positive values indicate a positive association between self‐enhancement and physical health. When studies reported effect sizes for multiple measures of self‐enhancement, physical health outcomes, groups or time points, we averaged these effects) so that each article contributed a single effect to the final model (*n* = 47). An analysis involving individual measures yielded virtually identical results. Most of the extracted effects were cross‐sectional (*n* = 77), but a few reflected prospective associations of self‐enhancement with health months or years later (ranging from 1 month to 10 years; *n* = 10).

The first and third authors coded an initial subset of studies to enable moderation tests and resolved disagreements by discussion (*n* = 65; all κ > .90). Specifically, we coded the measure of *self‐enhancement* as reflecting narcissism, optimistic bias, social desirability or other (i.e. illusory self‐beliefs, self‐superiority beliefs, subjective age bias). We also coded the specific *self‐enhancement scale* used in each study as a balanced inventory of desirable responding (BIDR; Paulhus, 1984), comparative judgement (ratings of self in comparison to others; Alicke & Govorun, 2005; Ziano et al., 2021), children's social desirability (CSD; Crandall et al., 1965), Marlowe‐Crowne social desirability (MCSD; Crowne & Marlowe, 1960; Reynolds, 1982), narcissistic personality inventory (NPI; Ames et al., 2006; Raskin & Terry, 1988) or other. We coded the *health outcome* examined in each study as biomarkers (e.g. blood pressure, heart rate, cortisol, telomere length), diseases (e.g. diabetes, HIV, cancer), obesity (e.g. BMI, weight loss), physical symptoms (e.g. pain, fatigue, health complaints, functional limitations), self‐rated health or other (included multiple health outcome types).

In terms of sample characteristics, we coded the *sample* as children, college students or other, the *sample region* as North America (Canada and USA, for these purposes) or other, and *sample health* as unhealthy (e.g. people with cancer, coronary heart disease, HIV, multiple sclerosis, obesity) or other. None of the physical health conditions were terminal. During effect size extraction, we recorded whether the *effect type* reflected a correlation or a between‐group analysis, where self‐enhancement or health was compared across select participant groups and the *effect time* as cross‐sectional or prospective. Finally, we extracted from studies, if applicable, the year of publication (*k* = 83), percentage of participants who were female (*k* = 79), percentage of participants in European American samples who were White (*k* = 38) and the mean age of participants (*k* = 73).

### Data analysis

We conducted a random‐effects meta‐analysis in R via packages such as *meta* and *metafor* (Schwarzer et al., 2015; Viechtbauer, 2010; see also Harrer et al., 2022). We searched for evidence of publication bias in the effect size distribution (i.e. selective publication of large or statistically significant effects) by adopting three strategies. First, we examined the distribution of obtained effect sizes in a funnel plot and used the Egger's test of the intercept (Egger et al., 1997) to evaluate whether the distribution was significantly asymmetrical, as would be expected if publication bias were present. Second, we used a trim‐and‐fill procedure to obtain a bias‐corrected estimate of the overall effect (Duval & Tweedie, 2000). Third, we used selection model analyses to estimate the overall effect after adjusting for potential publication bias through weight‐function modelling (Coburn & Vevea, 2019; McShane et al., 2016). We interpreted effect sizes (*r*s) using standard conventions (.10 = small, .30 = medium, .50 = large; Cohen, 1988).

## RESULTS

### Overall effect

#### Primary model

After aggregating across 87 independent studies, the overall association between self‐enhancement and physical health was near‐zero and not statistically significant, *r* = .01, 95% CI [−0.03, 0.04], *p* = .765 (Table 1). Nonetheless, there was considerable variability in the size of this association across studies, *Q* = 593.86, *τ* ^2^ = 0.03, *I* ^2^ = 85.5, which called for moderation tests exploring the conditions under which it was most pronounced.

**TABLE 1:** Estimates of overall effect size

Estimate type	*k*	*r*	95% CI
Primary model			
Random effects	87	.01	[−0.03, 0.04]
Sensitivity analyses			
Fixed‐effect	87	.03	[0.02, 0.04]
Unweighted	87	−.001	[−0.04, 0.04]
Publication bias‐corrected estimates			
Trim‐and‐fill	100	.05	[0.01, 0.10]
Weight function (moderate 2‐tail)	87	.01	–
Weight function (moderate 1‐tail)	87	−.04	–
Weight function (severe 2‐tail)	87	.01	–
Weight function (severe 1‐tail)	87	−.13	–

*Note*: *N* = 22,415. Weight‐function models do not provide 95% CIs.

#### Sensitivity analyses

We conducted follow‐up analyses to find out whether the magnitude of the association between self‐enhancement and physical health was influenced by assumptions of the primary model. Along these lines, a fixed‐effect analysis yielded a statistically significant association between self‐enhancement and physical health, but this effect was once again extremely small, *r* = .03, 95% CI [0.02, 0.04], *p* < .001. Further, an unweighted model that simply took the average of each effect regardless of its respective sample size (Bonett, 2009; Shuster, 2014) yielded an effect that was similar to the primary model and not statistically significant, *r* = −.001, 95% CI [−0.04, 0.04], *p* = .972. Taken together, these results indicate that the near‐zero association found between self‐enhancement and physical health was largely robust to different statistical approaches.

#### Publication bias

**FIGURE 2:**
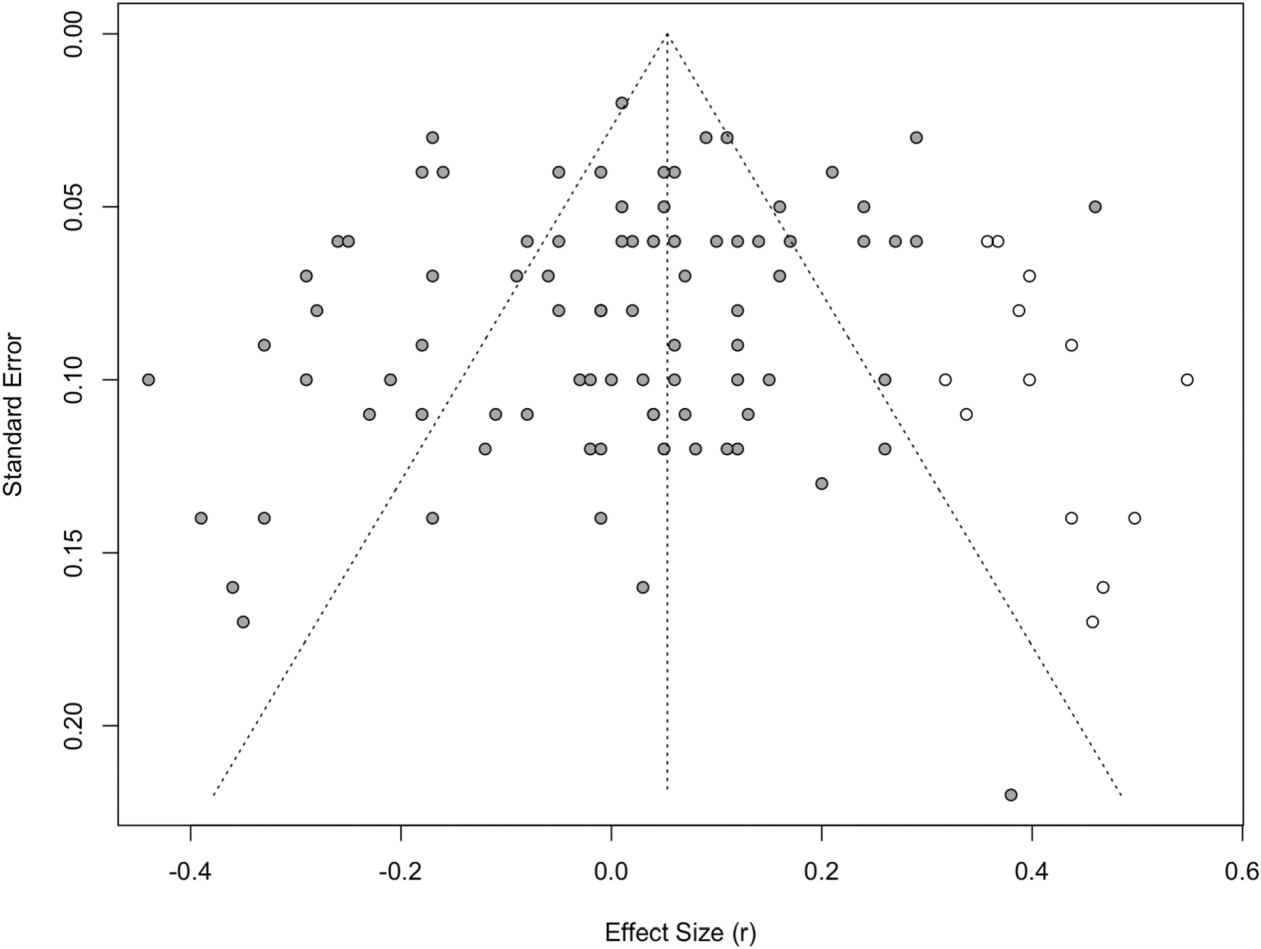
Funnel plot of effect sizes*. Note*: Dark circles are obtained effect sizes (*k* = 87), and light circles are effect sizes added via trim‐and‐fill (*k* = 13)

We used several strategies to evaluate the degree to which publication bias may have influenced our estimate of effect size. First, Egger's test was not statistically significant, intercept = −0.94, *p* = .122, suggesting that the distribution of effects was largely symmetrical. Further, a bias‐corrected (trim‐and‐fill) estimate of effect size was slightly larger than that obtained in the primary model and had a 95% CI that excluded zero, *r* = .05, 95% CI [0.01, 0.10], *p* = .014, 13 studies added (Figure 2), suggesting a slight bias in the literature toward publishing studies that evidenced a negative association between self‐enhancement and physical health. Nonetheless, even after adjusting for this bias, the overall association between self‐enhancement and physical health was close to zero.

Next, we quantified the possible role of publication bias via selection model analyses. Specifically, we used the weight‐function model to adjust for potential selection bias (Vevea & Hedges, 1995). When specifying *p*‐value cutpoints of .01, .05 and .10, there was no significant difference in effect size for the unadjusted model versus the adjusted model, Χ^2^(3) = 2.67, *p* = .445. In addition, we used selection model analyses to estimate the association between self‐enhancement and physical health assuming varying degrees of selection bias (Vevea & Woods, 2005). Selection model analyses produced adjusted estimates of effect size that were similar to the unadjusted estimate from the primary model, with the exception of the severe one‐tailed model (i.e. the most stringent test of publication bias), where we observed a small‐to‐medium inverse association between self‐enhancement and physical health (*r* = −.13). Altogether, the selection model analyses provide further evidence that publication bias did not have an undue influence on our primary estimate of effect size.

There are limitations with each of the reported publication bias tests, especially when effect size estimates are highly variable across studies (Harrer et al., 2022; McShane et al., 2016). For example, Egger's test may be somewhat insensitive to the detection of publication bias, trim‐and‐fill may insufficiently correct effect size estimates for publication bias, and selection model analyses rely on somewhat arbitrary *p*‐value cutpoints. Nonetheless, many of the obtained effect sizes were very small. Specifically, 27 studies reported an effect that was close to zero (absolute *r*s from .00 to .05) and 14 studies reported an effect that was very small (absolute *r*s from .06 to .10). Further, by adding unpublished effects (4) and effects that were not originally reported in published studies (13), we minimized the potential influence of publication bias on our overall estimate of effect size.

### Moderation tests

#### Self‐enhancement indices

The association between self‐enhancement and physical health did not vary significantly across different conceptualizations of self‐enhancement, *Q*(3) = 4.73, *p* = .193 (Table 2). That is, effect sizes were near‐zero when examining studies on social desirability (*r* = .03, *k* = 54), narcissism (*r* = −.06, *k* = 16) and optimistic bias (*r* = −.06, *k* = 9), with a majority of studies focussing on social desirability (62%). Conversely, effect sizes did vary significantly across the specific scales used to measure self‐enhancement, *Q*(5) = 27.06, *p* < .001. The most frequently used scales were the MCSD (*r* = .03, *k* = 41) and the NPI (*r* = −.0001, *k* = 8), which both yielded near‐zero effects. Other social desirability scales, that is the BIDR (*r* = .11, *k* = 4) and CSD (*r* = −.07, *k* = 4), yielded small effects. The largest effect emerged for comparative judgement, which yielded a small‐to‐medium association with health (*r* = .18, *k* = 6). These results indicate that the association between self‐enhancement and health varies across different indices of biased self‐views.

**TABLE 2:** Effect sizes across moderators

Moderator	*k*	*N*	*r* [95% CI]	*Q*	*Q*_btw_
Self‐enhancement					
Narcissism	16	2915	−0.06 [−0.13, 0.02]	53.2	4.7
Optimistic bias	9	2644	−0.06 [−0.24, 0.13]	106.1	
Other	8	1731	0.06 [−0.12, 0.25]	36.2	
Social desirability	54	15,125	0.03 [−0.02, 0.07]	345.4	
SE scale					
BIDR	4	1669	0.11 [−0.05, 0.27]	7.7	27.1**
CJ	6	1398	0.18 [0.07, 0.30]	13.3	
CSD	4	1188	−0.07 [−0.27, 0.13]	14.9	
MCSD	41	11,417	0.03 [−0.03, 0.08]	292.7	
NPI	8	1790	−0.0001 [−0.14, 0.14]	19.6	
Other	24	4953	−0.08 [−0.15, −0.01]	121.4	
Health outcome					
Biomarkers	17	1536	−0.13 [−0.22, −0.04]	48.1	19.2*
Diseases	8	2625	0.02 [−0.19, 0.22]	161.0	
Obesity	12	2834	0.01 [−0.06, 0.09]	25.2	
Other	12	4781	0.08 [0.02, 0.14]	24.9	
Self‐rated health	9	4873	0.09 [−0.01, 0.19]	82.3	
Symptoms	29	5766	0.01 [−0.06, 0.07]	175.1	
Sample type					
Children	5	1491	−0.02 [−0.22, 0.17]	27.1	0.4
College students	22	3892	−0.01 [−0.08, 0.07]	102.5	
Other	60	17,032	0.01 [−0.03, 0.06]	460.3	
Sample health					
Other	70	19,645	0.003 [−0.04, 0.04]	500.4	0.1
Unhealthy	17	2770	0.02 [−0.08, 0.11]	83.8	
Region					
North America	58	14,202	−0.01 [−0.06, 0.03]	347.1	1.8
Other	29	8213	0.04 [−0.02, 0.10]	246.8	

Abbreviations: BIDR, balanced inventory of desirable responding; CJ, comparative judgement; CSD, children's social desirability; MCSD, Marlowe‐Crowne social desirability; NPI, narcissistic personality inventory; SE, self‐enhancement.

* *p* < .05.

** *p* < .001.

#### Health outcome types

We observed significant variability in effect size magnitude across the six health outcome types, *Q*(5) = 19.21, *p* = .002. Specifically, there was a small positive association between self‐enhancement and self‐rated health (*r* = .09, *k* = 9) as well as between self‐enhancement and multiple health outcome types (*r* = .08, *k* = 12). However, there was a near‐zero association between self‐enhancement and diseases (*r* = .02, *k* = 8), obesity (*r* = .01, *k* = 12), and symptoms (*r* = .01, *k* = 29), in addition to a small negative association between self‐enhancement and biomarkers (*r* = −.13, *k* = 17). These results indicate that the association between self‐enhancement and physical health varies across health outcome types and is especially pronounced for self‐rated health.

#### Sample characteristics

Effect sizes did not vary significantly across different types of samples, *Q*s <1.9. That is, effects were near‐zero when examining children (*r* = −.02, *k* = 5), college students (*r* = −.01, *k* = 22) and other samples (*r* = .01, *k* = 60). Similarly, results did not vary when comparing physically unhealthy samples (*r* = .02, *k* = 17) to samples that were not restricted by health (*r* = .003, *k* = 70) and when comparing North Americans (*r* = −.01, *k* = 58) to people in other regions (*r* = .04, *k* = 29). The samples collected outside North America (i.e. Canada, USA) were derived from Europe (*k* = 17), New Zealand and/or Australia (*k* = 7), Israel (*k* = 2), in addition to China, Singapore and Uganda (*k* = 1 for each country). Finally, meta‐regression analyses indicated that effect sizes were not significantly associated with the gender, age, race‐ethnicity or year in which the study was published (*p*s > .182; Table 3).

**TABLE 3:** Meta‐regression analyses for continuous moderators

Moderator	*k*	*n*	*B* (*SE*)	*p*
Publication year	83	21,626	0.001 (0.002)	.465
% Female	79	21,189	0.0002 (0.0007)	.819
Age (mean)	73	20,510	0.0003 (0.001)	.787
% White	38	10,766	−0.002 (0.001)	.183

#### Methodological characteristics

We also found little variation in results across three methodological moderators, *Q*s <1.3 (Table 4). First, effect sizes were near‐zero both in studies that examined the correlation between self‐enhancement and physical health (*r* = .02, *k* = 58) and in studies that compared self‐enhancement or physical health across select participant groups (*r* = −.02, *k* = 29). Second, effect sizes varied little across cross‐sectional (*r* = .003, *k* = 77) and prospective analyses (*r* = .02, *k* = 10), while noting that the large majority of effects were cross‐sectional. Third, effect sizes were similar when comparing studies that provided a single effect (*r* = .02, *k* = 40) versus studies that provided multiple effects (*r* = −.01, *k* = 47). Thus, averaging effects within studies before entry into the model did not have an undue influence on our results.

**TABLE 4:** Effect sizes across methodological moderators

Moderator	*k*	*N*	*r* [95% CI]	*Q*	*Q*_btw_
Effect type					
Between‐groups	29	6508	−0.02 [−0.10, 0.05]	258.9	1.2
Correlation	58	15,907	0.02 [−0.02, 0.06]	285.1	
Effect time					
Cross‐sectional	77	20,412	0.003 [−0.04, 0.04]	535.1	0.2
Prospective	10	2003	0.02 [−0.09, 0.13]	43.2	
Effect count					
Multiple effects	47	10,891	−0.01 [−0.06, 0.04]	269.3	0.7
Single effect	40	11,524	0.02 [−0.03, 0.08]	311.1	

*Note*: Multiple effects = study provided multiple effects that were averaged before entry into the model; Single effect = study provided only a single relevant effect size.

## GENERAL DISCUSSION

We asked whether self‐enhancement conduces to physical health and reported the first meta‐analysis of self‐enhancement’s association with physical health, a critical outcome that underlies daily functioning and longevity. After aggregating across 87 studies, which included 22,415 participants, the overall association of self‐enhancement with physical health was near‐zero, with little direct evidence of publication bias. Furthermore, although moderation tests suggest that this association is influenced by the type of self‐enhancement and physical health measure implicated, the near‐zero effect size we obtained was generally robust to different methodological factors and sample characteristics.

### Implications

Our meta‐analysis makes several contributions to the self‐enhancement literature. First, by uniquely focussing on physical health and aggregating across a large and diverse set of studies, we provided a comprehensive estimate of the adaptiveness of self‐enhancement. We showed that the overall association of self‐enhancement with physical health is rather negligible. Self‐enhancement does not appear to reap substantial physical health benefits.

Second, we demonstrated that the association between self‐enhancement and physical health fluctuates across measures of both constructs. Self‐enhancement yielded a small positive association with self‐rated health, but this association was likely inflated by common method variance (i.e. both measures were obtained from the same source; Strickhouser et al., 2017) or the possibility that self‐enhancement contaminates ratings of one's health. Consistent with this argument, self‐enhancement yielded a near‐zero association with other health outcomes, such as diseases, symptoms and obesity, and indeed yielded a small negative association with biomarkers that was statistically significant (i.e. had a 95% confidence interval that excluded 0). Thus, physical health outcomes assessed via self‐reports (self‐rated health) yielded positive associations with self‐enhancement, but physical health outcomes that are assessed more objectively (diseases, biomarkers) yielded near‐zero or even negative associations.

Results were somewhat inconsistent when examining the different conceptualizations and measures of self‐enhancement. In support of the argument that different conceptualizations of self‐enhancement reflect the same higher‐order construct (Sedikides, 2021b; Sedikides & Gregg, 2008; Taylor & Brown, 1988), we found no significant difference in effect size across these concepts (i.e. narcissism, optimistic bias, social desirability). However, when zeroing on the specific scale used to measure self‐enhancement, we found significant fluctuations in effect size. Frequently used measures such as the MCSD and NPI yielded near‐zero associations with physical health, but the BIDR yielded a small positive association with physical health and comparative judgements yielded a small‐to‐medium positive association with physical health. Although these results should be interpreted with caution due to a relatively small number of effect sizes for some scales (*k* < 9), they provide preliminary evidence that associations of self‐enhancement with physical health are more detectable when measured via some scales than others. Comparative judgement and BIDR may be more direct measures of self‐enhancement than the MCSD and NPI, which appear to involve other constructs in addition to self‐enhancement (e.g. concern for social approval, global self‐esteem, status).

Third, unlike the significant moderation that we observed for measures of self‐enhancement and physical health, effect sizes were largely constant across a variety of sample and methodological characteristics. Along these lines, the association between self‐enhancement and physical health was negligible across age, gender, race‐ethnicity and country. In addition, we obtained near‐zero effects regardless of whether prior studies used correlational or between‐subjects designs and whether they reported cross‐sectional or prospective associations between self‐enhancement and physical health. Taken together, these results indicate that the near‐zero association between self‐enhancement and physical health is largely robust across different samples and methods examined in the literature so far.

Fourth, and more broadly, our meta‐analysis synthesized research on self‐enhancement and physical health across several disciplines, including social, personality, health, clinical and biological psychology as well as public health, medicine and sociology. Prior reviews of this topic were qualitative and focussed on only a portion of the available research literature (Konrath & Bonadonna, 2014; Segerstrom & Roach, 2008; Taylor & Sherman, 2008). In our comprehensive analysis, we found that studies have used a wide variety of measures, samples and research practices. We found little direct evidence of publication bias, with many published effects being close to zero. Nonetheless, we observed substantial variability in effect sizes, with associations between self‐enhancement and physical health ranging from medium‐to‐large negative effects to large positive effects (Figure 2). Taken together, our results indicate that the effects of self‐enhancement are variable and context dependent.

### Limitations and future directions

Although our meta‐analysis provides a comprehensive estimate of the association between self‐enhancement and physical health, limitations necessitate additional research. Most of the included studies that we obtained used socially desirable responding as the measure of self‐enhancement (*k* = 54; 62%). More importantly, of the 87 effect sizes, 41 (47%) were derived from studies that examined the relation between the MCSD and physical health markers. This may be problematic. First, the MCSD is not widely regarded as a hallmark index of self‐enhancement. The scale is very similar to the BIDR's impression management subscale; so, the MCSD may assess more closely other‐deceptive, rather than self‐deceptive, self‐enhancement: It may predominantly capture style rather than substance (bias). To the extent that it assesses substance, the scale may be pertinent to defensiveness or neuroticism (Andrews & Meyer, 2003; McCrae & Costa, 1983; Weihs et al., 2000), tapping self‐protection (Sedikides, 2012; vanDellen et al., 2011) as opposed to self‐enhancement strivings. Finally, the MCSD, despite its overall adequacy, has met with some criticism regarding its validity (Ballard, 1992; Ballard et al., 1988) and reliability (Beretvas et al., 1992; O'Grady, 1988). Of note, the other operationalisation of social desirability, the BIDR, evinced a positive association with physical health (*r* = .11) and was stronger than that of the MCSD; thus, the BIDR may be a better proxy for self‐enhancement than the MCSD.

Comparative judgement yielded a small‐to‐medium positive association with physical health across six studies. Follow‐up work could derive robust estimates of effect size for other, more direct indices of self‐enhancement. A review published in 2010 identified 60 of such indices (Hepper et al., 2010) and since then more than a dozen additional indices have been documented (Sedikides, 2020). Some examples include favourable interpretation of ambiguous feedback, selectively approaching individuals who are likely to deliver positive feedback, assuming credit for the successes of the dyad or group, comparing favourably the present self with the past self, ‘holier than thou’ perceptions, and resorting to counterfactual thinking.

We located only a small number of studies in conjunction with each physical health outcome. Follow‐up work could clarify the nature and robustness of the association of self‐enhancement with each of these specific outcomes, particularly biomarkers, which yielded a small negative association across 17 studies, and self‐rated health, which yielded a small positive association across nine studies.

Our meta‐analysis was also limited to the inclusion of studies published in English. Very few studies examine associations of self‐enhancement with physical health outside of Western societies (*k* = 3; 3%). Research in other countries and cultures is needed to assess whether the near‐zero association of self‐enhancement with physical health is universal (Church et al., 2014). Moreover, as the current meta‐analysis found that most studies in this literature are cross‐sectional, longitudinal studies are needed to test whether self‐enhancement is associated with changes in physical health across time. Assuming they exist, small effects of self‐enhancement on physical health may take years or even decades to manifest. Thus, research testing whether self‐enhancement in adolescence predicts later physical health would be especially informative (Steiger et al., 2014). Research examining associations of self‐enhancement with health behaviours (Davidson & Prkachin, 1997), such as eating, exercise and sleep, is also necessary to test the adaptiveness of self‐enhancement and expand the literature.

Given that this meta‐analysis yielded considerable heterogeneity in associations between self‐enhancement and physical health, much of it unexplained, primary research is needed to uncover additional moderators. Such research should test whether associations vary across specific aspects or sub‐components of self‐enhancement. Evidence indicates that psychological health is more strongly associated with (a) inflated views of one's social skills than inflated views of one's intelligence (communal vs. agentic narcissism; Rentzsch & Gebauer, 2019), (b) self‐promoting aspects of narcissism (grandiosity) than defensive aspects (hypersensitivity; Edelstein et al., 2012) and (c) perceiving as opposed to merely presenting the self in a positively biased manner (Paulhus, 2002). Thus, it is possible that some aspects of self‐enhancement are more strongly associated with physical health than others. Moreover, the present meta‐analysis suggests that self‐enhancement may have both positive and negative pathways to health (that cancel each other out) or may have no association with health (Esterhuizen & Thabane, 2016). Future work would do well to test possible pathways and the conditions under which they occur.

Another consideration for follow‐up research is the specificity or match between measures of self‐enhancement and physical health. As of present, studies have primarily examined the association between general measures of self‐enhancement across domains and specific health outcomes (Taylor et al., 2003). However, the association between attitudes and behaviour is more pronounced when measures of attitudes and behaviour are matched in their specificity (Ajzen & Fishbein, 2005). Further, although global self‐esteem often fails to predict specific behaviours, specific self‐concepts are more predictive of these behaviours (Swann Jr. et al., 2007). Thus, future work should examine whether specific indices of self‐enhancement (e.g. overestimations of one's cardiovascular or metabolic health) predict relevant physical health outcomes over time (e.g. heart disease or diabetes). Researchers should also use measures of self‐enhancement at the individual level (e.g. narcissism, social desirability) either instead of or in addition to measures of self‐enhancement at the aggregate level (e.g. above average effects), given that the former is better matched to health outcomes, which are also assessed at the individual level, than the latter.

### Conclusions

The question of whether self‐enhancement conduces to psychological health has stimulated scholarship for over 35 years, and it has been answered in the affirmative. The present meta‐analysis is the first to examine the question of whether self‐enhancement conduces to physical health, arriving at a contingent answer. Although the overall association between self‐enhancement and physical health was near‐zero, this association was more pronounced for comparative judgement than social desirability or narcissism, and for self‐rated health than symptoms or biomarkers. Follow‐up investigations are needed to clarify the precise conditions under which self‐enhancement reaps benefits (or costs, as it may) for physical health, in addition to whether these effects manifest over time. Although much knowledge has been accumulated, many questions remain about the granular implications of self‐enhancement for physical health, awaiting the next generation of scholars.

## CONFLICT OF INTEREST

The author declares that there is no conflict of interest.

## Data Availability

A complete dataset and list of included studies in the meta‐analysis are publicly available on OSF at https://osf.io/tpzgv/?view_only=b35ffe3a903d44f381c129712e605fd8
